# PBC, an easy and efficient strategy for high-throughput protein C-terminome profiling

**DOI:** 10.3389/fcell.2022.995590

**Published:** 2022-08-31

**Authors:** Linhui Zhai, Le Wang, Hao Hu, Quan Liu, Sangkyu Lee, Minjia Tan, Yinan Zhang

**Affiliations:** ^1^ School of Chinese Materia Medica, School of Pharmacy, Nanjing University of Chinese Medicine, Nanjing, Jiangsu, China; ^2^ Jiangsu Key Laboratory for Functional Substances of Chinese Medicine, School of Pharmacy, Nanjing University of Chinese Medicine, Nanjing, Jiangsu, China; ^3^ State Key Laboratory of Drug Research, Shanghai Institute of Materia Medica, Chinese Academy of Sciences, Shanghai, China; ^4^ College of Pharmacy and Research Institute of Pharmaceutical Sciences, Kyungpook National University, Daegu, South Korea

**Keywords:** C-terminomics, chemical derivatization, enrichment, high-efficiency, post-translation modification (PTM)

## Abstract

High-throughput profiling of protein C-termini is still a challenging task. Proteomics provides a powerful technology for systematic and high-throughput study of protein C-termini. Various C-terminal peptide enrichment strategies based on chemical derivatization and chromatography separation have been reported. However, they are still costly and time-consuming, with low enrichment efficiency for C-terminal peptides. In this study, by taking advantage of the high reaction selectivity of 2-pyridinecarboxaldehyde (2-PCA) with an α-amino group on peptide N-terminus and high affinity between biotin and streptavidin, we developed a 2-PCA- and biotin labeling–based C-terminomic (PBC) strategy for a high-efficiency and high-throughput analysis of protein C-terminome. Triplicates of PBC experiments identified a total of 1,975 C-terminal peptides corresponding to 1,190 proteins from 293 T cell line, which is 180% higher than the highest reported number of C-terminal peptides identified from mammalian cells by chemical derivatization–based C-terminomics study. The enrichment efficiency (68%) is the highest among the C-terminomics methods currently reported. In addition, we not only uncovered 50 proteins with truncated C-termini which were significantly enriched in extracellular exosome, vesicle, and ribosome by a bioinformatic analysis but also systematically characterized the whole PTMs on C-terminal in 293 T cells, suggesting PBC as a powerful tool for protein C-terminal degradomics and PTMs investigation. In conclusion, the PBC strategy would benefit high-efficiency and high-throughput profiling of protein C-terminome.

## Introduction

Protein N-termini and C-termini play important roles in diverse biological processes such as protein stability, protein localization, protein–protein interaction, and macromolecular complexes formation (Marino et al., 2015; Perrar et al., 2019; Winter et al., 2021). The high-throughput study of protein termini and their posttranslational modifications (PTMs) is important for understanding their functions. (Marino et al., 2015; Klein et al., 2018; Chen and Kashina, 2021; Chi et al., 2021). Proteomics technologies have emerged as a powerful tool for the systematical and high-throughput analysis of protein termini. Thus far, various strategies have been developed for protein termini enrichment (Huesgen and Overall, 2012; Rogers and Overall, 2013; Koudelka et al., 2021). However, current methods for protein C-terminome profiling still lag far behind the N-terminomics technologies. More than 7,400 N-terminal peptides were reported to be identified without pre-fractionation in human lymphoblastoid B cell line (Klein et al., 2015), while the reported highest number of identified C-terminal peptides was 3,129 with the requirement of extensive off-line HPLC fractionation (24 fractions) in HeLa cells. (Wang et al., 2021) There are several technical difficulties leading to the lower coverage of C-terminome than that of N-terminome. First, it was reported that more than 60% human protein lack lysine or arginine residues near C-termini (Wang et al., 2021). For these proteins, the C-terminal peptides generated by the widely used proteases in proteomics study (i.e., trypsin and LysC) do not contain appropriate length for efficient LC-MS/MS detection. Second, the lack of basic amino acid residue will affect the ionization efficiency in positive mode for mass spectrometry detection. Third, because of the low chemical reaction reactivity of the C-terminal α-carboxyl group, the development of chemical derivatization–based C-terminal enrichment approach is largely restricted.

Current C-terminal peptide enrichment strategies are mainly classified into two different types. One is chromatography-based C-terminal peptide direct enrichment. This strategy is based on the physiochemical difference between C-terminal peptides and other internal peptides, such as the hydrophilic/hydrophobic properties and isoelectric point (Dormeyer et al., 2007; Van Damme et al., 2010; Wang et al., 2021). In order to increase the physiochemical difference between the C-terminal peptides and non-C-terminal peptides, the amidation of carboxyl group on protein C-terminal or propionylation of amino group on peptide was performed prior to chromatographic separation (Kaleja et al., 2019; Li et al., 2020a). Though the chromatography-based strategy provides an easy way for C-terminal peptides enrichment, the selectivity and efficiency of these methods are still not satisfactory, due to the low separation resolution for highly complex peptide mixtures. This strategy also suffers from intensive labor and instrumentation cost, which requires lots of off-line prefractions and high MS instrument time.

Another C-terminal peptide enrichment strategy is the chemical derivatization–based negative enrichment method. This strategy is based on chemical derivatization to protect carboxyl group on original protein C-terminal at the protein level; then, proteins were digested into peptides and the internal peptides with free carboxyl group were removed by the polyallylamine polymer. The C-TAILS (C-terminal amine–based isotope labeling of substrate) method was first reported for C-terminal peptide enrichment by Schilling et al. (2010). In this method, the α-amine groups on protein N-termini were first blocked by dimethylation, and the carboxyl groups on protein C-termini were then blocked by ethanolamidation. After the digestion of proteins with trypsin, the neo-α-amine groups exposed from the internal peptides were further blocked by dimethylation. Finally, the neo-internal peptides containing free carboxyl groups were coupled and depleted with the poly(allylamine) polymer. The C-terminal peptides were then enriched. After the introduction of the C-TAILS method, different kinds of C-TAILS–based methods were further developed. Zhang et al. (2015) used Ac-NHS to block the α-amine group on the protein level and used ethanolamine to block the carboxyl group before using a high molecular polymer to negatively enrich C-terminal peptides. This method achieved a higher yield of chemical derivatization and identified more C-terminal peptides than the original C-TAILS method. We developed the LAACTer method on the basis of C-TAILS (Hu et al., 2019). Our LAACTer method combined LysargiNase digestion, chemical reaction, and ion-aided proteome database searching for an in-depth C-terminomic study and finally identified 164% and quantified 300% more C-terminal peptides than those using the original C-TAILS method from 293 T cells.

Although the reported chemical derivatization–based C-terminome methods provide powerful technologies to systematically study the C-terminal peptides, most of them requires at least three steps of chemical reaction on protein and peptide levels; thus, they are labor- and time-consuming. The amidation on the carboxyl group used in these methods are largely low-specific, which influences the C-terminal peptides enrichment efficiency (Zhang et al., 2015). In addition, the LysargiNase used in the LAACTer method is expensive and not conventionally used in the proteomics study. So far, a high-efficiency and high-throughput analysis of C-terminal peptides is still challenging. In order to make the chemical derivatization–based strategies more practical and efficient, we developed a new method, namely, 2-pyridinecarboxaldehyde (2-PCA)- and biotin labeling–based C-terminomics (PBC), for high-throughput and highly efficient enrichment of C-terminal peptides. In this study, we found that the peptide length and hydrophilic properties could significantly affect the 2-PCA labeling efficiency on the α-amine group. Through the PBC method, we obtained the highest number of C-terminal peptides and highest enrichment efficiency in chemical derivatization–based C-terminomics up to date. We also systematically revealed the PTMs on C-terminal peptides and C-terminal truncated proteins in 293 T cells by combined usage of the PBC strategy and an open-search method. In this regard, our newly developed PBC method provides a powerful tool to efficiently study the C-terminome from a low amount of samples.

## Methods

### Cell culture

The human embryonic kidney cell line HEK 293 T was cultured in Dulbecco’s modified Eagle’s medium (DMEM). After the cells grew to 80% density in a 10-cm petri dish, the medium was washed out and the cells were harvested by centrifugation at 1,000 *g* for 5 min under room temperature. Then the cell pellets were washed with cold PBS buffer twice.

### Proteome sample preparation

HEK 293 T cell pellets were suspended in lysis buffer [6 M guanidine hydrochloride, 100 mM HEPES (pH 8.0), and 1% (v/v) protease inhibitor cocktail (Roche, Swiss)] on ice for 30 min, followed by sonication for 2 min with 2 s on and 5 s off at 30% power. Then the lysates were centrifuged at 20,000 g at 4°C for 10 min and the supernatant was collected. Protein concentration was measured by using the BCA assay (Beyotime, China). For PBC technical evaluation, the 900 µg extracted proteins were used and divided into three equal parts (300 µg protein/each part). The proteins were reduced by using 5 mM dithiothreitol (DTT) at 56°C for 30 min and alkylated by using 15 mM iodoacetamide (IAA) in darkness at 25°C for 30 min; 20 mM DTT was added to the protein solution to quench the excess IAA. Then the proteome sample was digested with LysC (Hualishi, China) with an enzyme/protein ratio of 1:50 (w/w) at 37°C overnight.

### 2-PCA labeling and sulfo NHS-biotin labeling

For 2-PCA labeling, 10 mM 2-PCA (J&K Scientific, China) dissolved in 100 mM HEPES buffer (pH 8.0) was added to the 300 µl peptide solution (300 µg peptide) at 37°C for 16 h (MacDonald et al., 2015) For sulfo NHS-biotin labeling, 2 mM sulfo NHS-biotin (APExBIO, United States ) dissolved in 100 mM HEPES (pH 8.5) was added to the 2-PCA–labeled peptide sample at 37°C for 30 min. The labeled-peptide samples were dried in SpeedVac and then desalted by using Sep-Pak C_18_ cartridges (50 mg sorbent per cartridge, Waters, United States ).

### Enrichment of C-terminal peptides

A volume of 1 ml streptavidin beads (GE Healthcare, United States) were washed twice with 800 µl PBS. The 2-PCA- and biotin-labeled peptide was re-suspended to 800 µl PBS, pH was adjusted to 7.5, and then it was incubated with streptavidin beads at room temperature for 1 h. The supernatant was collected by centrifugation at 300 g for 2 min, and the beads were washed with 800 µl PBS twice. The supernatant and washing solution was combined and dried in SpeedVac.

### C-terminal peptides fractionation and desalting

The C-terminal peptides were fractionated by using a home-made StageTip C_18_ column. The StageTip C_18_ column was made as follows. C_18_ disks (3M, United States ) were cut by a hypodermic needle and pushed into P200 pipet tips. Then 2mg C_18_ resin (Durashell C_18_, Agela, China) was re-suspended in 200 µl acetonitrile (ACN), loaded into prepared pipet tips, and then centrifuged at 400 g for 10 min. The StageTip column was equilibrated with 150 µl water of 0.1% ammonium hydroxide (NH_3_·H_2_O) for three times, respectively; the centrifuge time was controlled in 10 min. Then the peptide sample was loaded onto the column, the column was washed by 150 µl water (0.1% NH_3_·H_2_O), and the peptides were eluted with 2%, 9%, 15%, 20%, 24%, 30%, and 80% ACN in water (0.1% NH_3_·H_2_O). The fractionations were dried in SpeedVac and desalted with ZipTip C_18_ (Millipore, United States).

### LC-MS/MS analysis

The sample was analyzed by using an EASY-nLC 1200 HPLC tandem with the Q Exactive HF-X mass spectrometer (Thermo Fisher Scientific, United States). The peptide was resolved in buffer A (2% ACN in water and 0.1% formic acid) and separated by using a home-made C_18_ capillary column (25 cm × 75 μm, 1.9 µm particle size, and 100 Å pore size) (Li et al., 2020b), A column oven was used and the heating temperature was set at 60°C (Kyte and Doolittle, 1982).

For PCA- and biotin-labeled evaluation, the peptide sample before and after labeling were detected by using 1 h gradient LC-MS/MS. The LC gradient was set as follows: 8 %–13% buffer B (90% ACN in water and 0.1% formic acid) for 20 min; 26% buffer B for 31 min, with a raise to 45% in 5 min; and finally 80% buffer B for 60 min. The flow rate was set to 300 nL/min. Then ions were scanned over 350–1,300 m/Z at a resolution of 12,000 (200 m/Z) with the automatic gain control (AGC) target of 5.0e5 and maximum injection time of 50 ms. The charge state included was 2–6 and dynamic exclusion was 60 s. The data-dependent mode was set up with a cycle time of 3 s, and MS2 data were acquired by higher-energy collisional dissociation (HCD) fragmentation and normalized collision energy (NCE) of 32%. The AGC target was set to 7.0e3 and maximum injection time was set to 35 ms.

The enriched C-terminal peptide was detected by 2 h gradient LC-MS/MS. The LC gradient was set as follows: 2%–5% buffer B for 3 min; to 16% buffer B for 52 min; to 35% buffer B for 50 min, with a raise to 47% for 10 min; and finally 80% buffer B for 120 min. The flow rate was set as 300 nL/min. Then peptides were scanned over 350–1,800 m/z at a resolution of 60,000 with the automatic gain control (AGC) target of 3e6 and maximum injection time of 45 ms. The charge state included was 1–5 and dynamic exclusion was 20 s. The data-dependent mode was set up with the top 10 most abundant precursors and subjected to MS/MS fragmentation, and MS2 data were acquired by higher-energy collisional dissociation (HCD) fragmentation and normalized collision energy (NCE) of 28%. The AGC target was set to 1e5 and the maximum injection time was set to 80 ms.

### Database searching and bioinformatic analysis

The raw data were searched against the UniProt homo proteome database (version 201,812) through Proteome Discoverer (version 2.2, Thermo Fisher Scientific) and loaded into the Mascot search engine (version 2.3, Matrix Science). The enzyme type was Lys-C/P. Up to two maximum missed cleavage was used. The precursor mass tolerance was set as 10 ppm, and the fragment mass tolerance was set as 0.02 Da. For samples before enrichment, carbamidomethyl (C) was set as static modification, and acetyl (protein N-term) and oxidation (M) were set as dynamic modifications. In addition, PCA (N-term), biotinylation (K), and biotinylation (N-term) were set as dynamic modifications for analyzing sulfo NHS-biotin–labeled samples. For sample of enrichment, carbamidomethyl (C) and biotinylation (K) were set as static modifications, and PCA (N-term) and oxidation (M) were set as dynamic modifications. The minimal peptide length was filtered with six amino acids. The results were filtered by a 1% false discovery rate (FDR) at PSM, peptide, and protein levels (Zhang et al., 2018; Wang et al., 2021).

For the open-search, all raw data files were processed using pFind software (version 3.1.0) with an open-search mode (Kahl et al., 2018). The enzyme type was set as Lys-C/P, and the maximum missed cleavage was 2. The precursor mass tolerance was 10 ppm and fragment mass tolerance was 10 ppm. The results were filtered by a 1% false discovery rate (FDR) at both PSM and protein levels (Chi et al., 2018; Guangcan et al., 2021).

The determination of 2-PCA and biotin labeling efficiency analyses and other character results were carried out with GraphPad (version 8.0). All statistical tests were analyzed using student *t*-tests. The GRAVY scores were calculated using the online tool (https://web.expasy.org/protparam/) (Kyte and Doolittle, 1982). The analysis of peptide sequences was conducted by iceLogo (Colaert et al., 2009), and a *p*-value < 0.05 was used. The bioinformatic analysis was performed using DAVID (version 6.8) with an adjusted *p*-value < 0.05 (Huang et al., 2007).

### Data availability

All the original proteomics raw data and proteome database result files in this study have been deposited to the iProX Consortium with the subproject ID IPX0003710000.

## Results and Discussion

### Strategy design for 2-PCA- and biotin labeling–based C-terminomic

**FIGURE 1:**
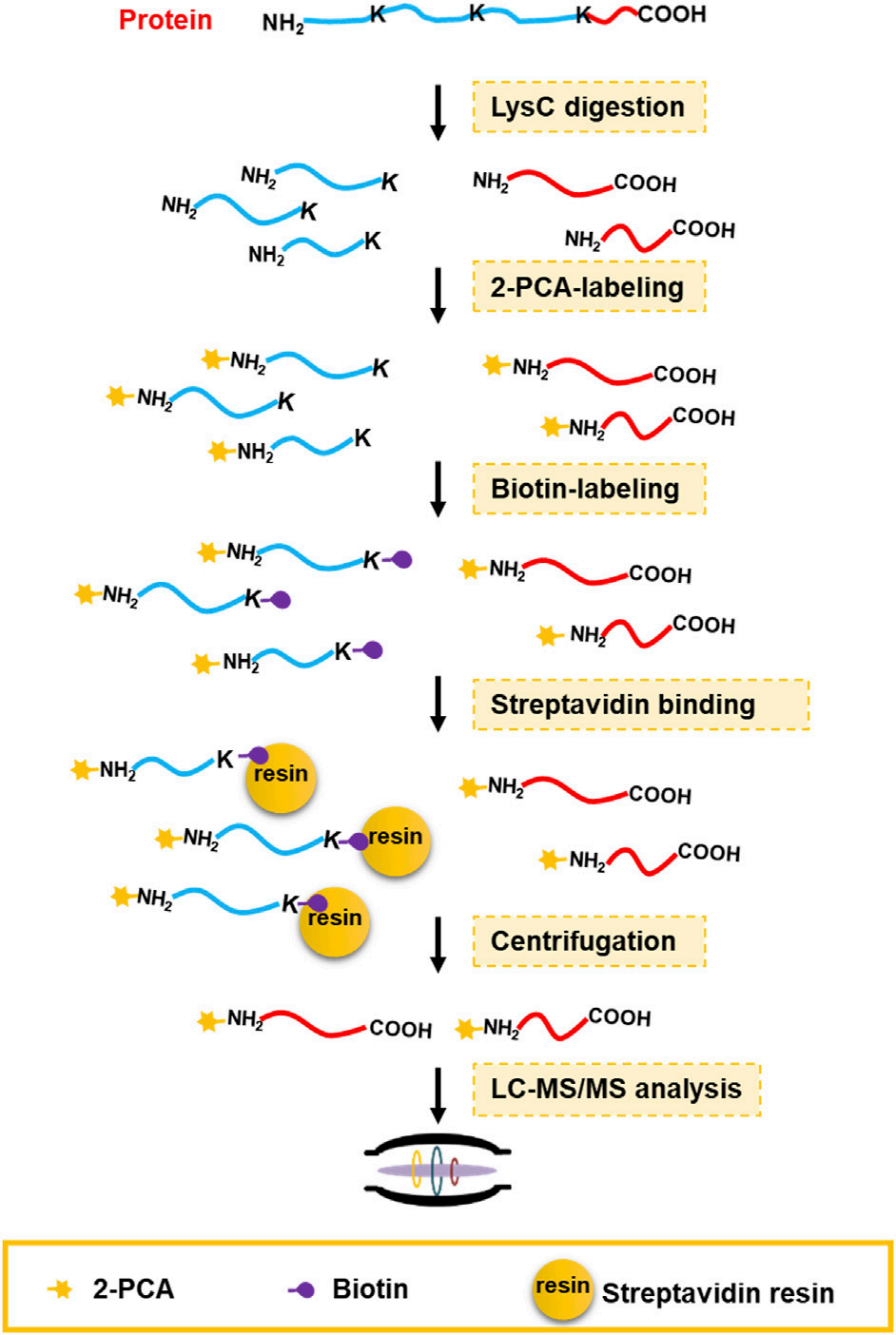
C-terminal peptide enrichment workflow through the PBC strategy. The extracted whole cell proteome is digested into peptides by LysC protease. Then the LysC-digested peptides are tandemly reacted with 2-PCA and NHS-biotin. Next, the peptides with biotin-labeled lysine were captured by streptavidin beads. Finally, the C-terminal peptides are enriched by filter-aided separation from streptavidin beads and detected by LC-MS/MS.

Chemical derivatization on protein or peptide is an enabling technology for a proteomics study. 2-PCA reported could selectively label the α-amino group on protein/peptide N-terminus over the ε-amino group of lysine residue through an N-terminal amine-specific cyclization reaction (MacDonald et al., 2015). By taking advantage of such reaction selectivity of 2-PCA, we designed a PBC strategy for high-throughput profiling of protein C-termini by bottom-up shotgun proteomics (Figure 1). The extracted whole cell proteome is digested into peptides by LysC protease, which cleaves peptide bond C-terminal to Lys residue. As a result, each non-C-terminal peptide ends with lysine and thus contains two amino groups on the peptide N-terminus and the lysine side chain. In contrast, C-terminal peptides lack lysine and only contain one α-amino group on peptide N-termini. Next, the peptides are tandemly reacted with 2-PCA and NHS-biotin. Ideally, all the α-amino groups on the peptide N-terminal are blocked with 2-PCA and ε-amino groups on the lysine side chain are labeled with biotin. The chemically derivatized peptides are then incubated with streptavidin beads. The original protein N-terminal peptides and internal peptides containing biotin-labeled lysine are all captured by streptavidin beads. Finally, the C-terminal peptides are enriched by filter-aided separation from streptavidin beads and detected by LC-MS/MS. Thus, our PBC strategy not only took advantage of LysC, which has higher digestion efficiency and lower cost than other commonly used proteases (LysargiNase and ArgC) (Giansanti et al., 2016) but also employed only two chemical derivatization steps at the peptide level, in contrast to other reported negative C-terminal peptide enrichment strategies requiring at least three chemical derivation steps at both protein and peptide levels (Schilling et al., 2010; Zhang et al., 2015; Hu et al., 2019). Therefore, the PBC strategy is less labor- and time-consuming for C-terminal peptide enrichment.

### 2-PCA could efficiently and highly selectively label the peptide N-terminal

In a previous study (MacDonald et al., 2015), only limited number of peptides (20 peptides) with similar sequence composition (only varying at N-terminal amino acid) were used to evaluate the characteristics of the reaction between 2-PCA and the peptide N-terminal α-amino group. Whether other factors could affect the selectivity and efficiency of this reaction needs to be deeply studied by using a large-scale and highly complex peptide sample. In this study, we first used the mass spectrometry–based shotgun proteomics approach to systemically study the characteristics of the selective reaction with the α-amino group on the peptide N-terminus. The highly complex peptides were generated by using LysC digested whole HEK 293 T cell proteome. The evaluation for 2-PCA labeling efficiency was performed in technical triplicates from the same proteome sample. The peptides were reacted with 2-PCA and then detected by LC-MS/MS. Compared to the unlabeled sample, the number of identified protein and peptide decreased in the 2-PCA–labeled sample under same LC-MS/MS conditions. We reasoned that the LC gradient for 2-PCA–labeled peptide sample was unsuitable and needed to be further optimized because the peptide retention time was changed after 2-PCA labeling (Supplementary Figures S1A,B; Supplementary Tables S1, S2). In order to evaluate whether 2-PCA could react with the ε-amino group on lysine, we both set the 2-PCA labeling at the peptide N-terminal and lysine as variable modification for database searching. The bioinformatic analysis results showed 2-PCA dominantly labeled at the peptide N-terminal, while only 2.3% lysines were labeled (Supplementary Figure S1C; Supplementary Table S3). Such results agreed with previously reported result that 2-PCA labeling could selectively react with the α-amino group on the peptide N-terminal. (MacDonald et al., 2015)

**FIGURE 2:**
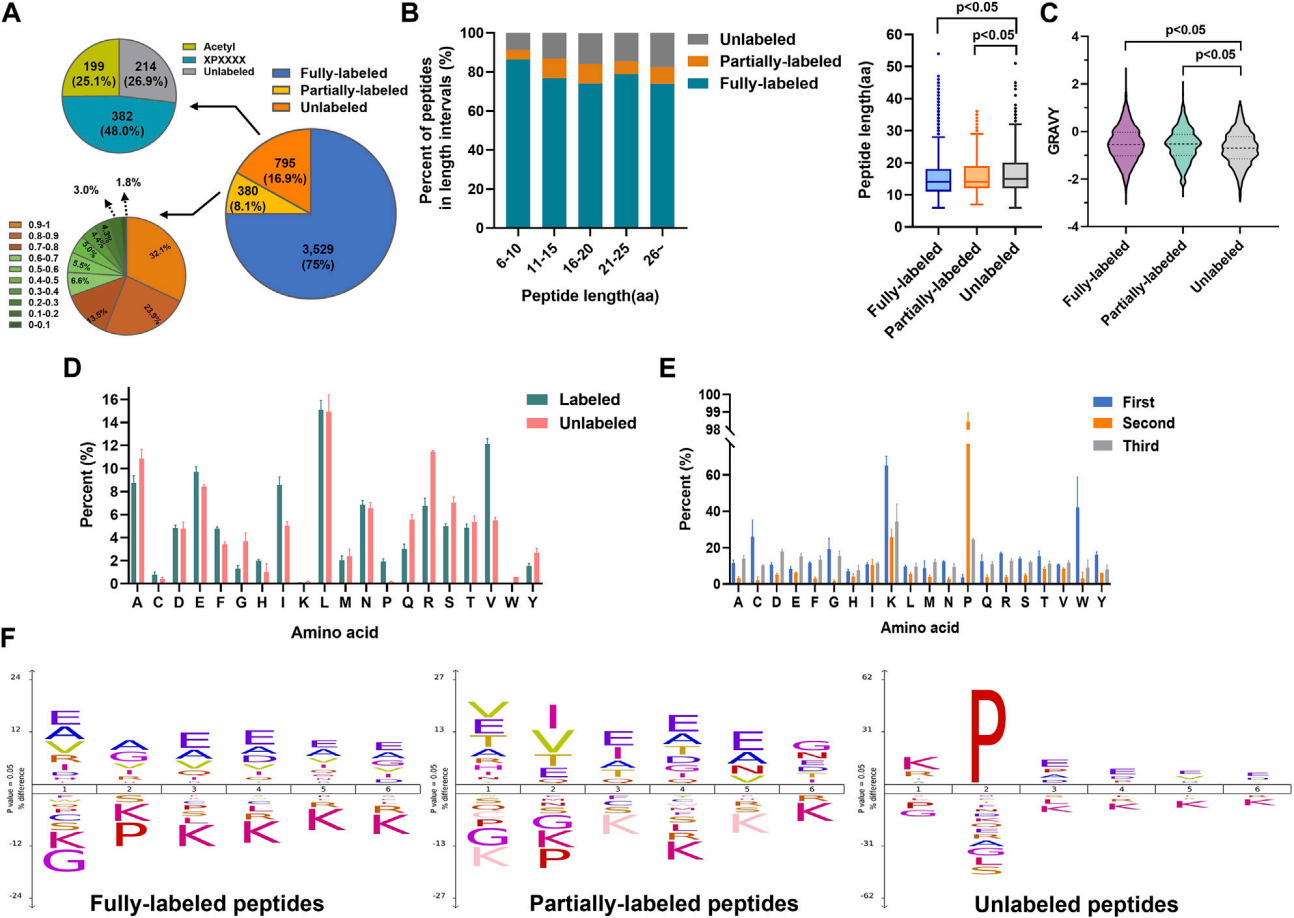
2-PCA displays efficiently and high selectively labeling peptide N-terminal. **(A)** Pie chart of the 2-PCA labeling efficiency distribution calculated by using the spectral counting-based quantification method. **(B,C)** 2-PCA labeling efficiency of different length **(B)** and hydrophilic/hydrophobic value of peptide **(C)**. The GRAVY scores were calculated using the online tool (https://web.expasy.org/protparam/) (Kyte and Doolittle, 1982). The *p*-value was calculated by paired Student *t*-test. **(D,E)** Full labeling **(D)** and un-labeling **(E)** peptide percent on different first three amino acids. **(F)** IceLogo analysis representation of the first six amino acids of full labeling, partial labeling, and un-labeling peptides.

Then we evaluated the N-terminal labeling efficiency (the percentage of 2-PCA–labeled peptides in all peptides). Among 4,704 identified non-redundant peptides (peptides of the same sequence with different 2-PCA modification status were considered as two different peptides), 75% peptides (3,529 peptides) were fully labeled with 2-PCA, 8.1% peptides (380 peptides) were partially labeled, and 16.9% peptides (795 peptides) were completely unlabeled (Figure 2A, Supplementary Table S2). For the 795 unlabeled peptides, 25.1% peptides (199 peptides) were N-terminally acetylated and 48.0% (382 peptides) were identified to possess proline at the second amino acid position. We used spectral counting–based quantification to calculate the N-terminal labeling efficiency for the 380 partially 2-PCA–labeled peptides and found 122 peptides (occupied 32.1%) with labeling efficiency higher than 90% and 187 peptides (occupied 49.4%) with labeling efficiency between 50% and 90%. As mentioned before, by excluding the N-acetylated and proline-containing peptides which could not react with 2-PCA in theory, we found more than 90.7% peptides were fully labeled or with labeling efficiency higher than 80%. Such results showed that 2-PCA could react with peptide N-terminal with high efficiency and high selectivity.

We next analyzed factors that influenced the 2-PCA-labeling efficiency, such as peptide length, hydrophilic/hydrophobic properties, and amino acids composition. As shown in Figure 2B, the peptide length significantly affected the 2-PCA–labeling efficiency, with short peptides more preferred. The length of unlabeled peptides was significantly longer than the fully labeled or partially labeled peptides. We also explored the relationship between peptide hydrophilicity (evaluated by GRAVY score) and labeling efficiency (Figure 2C). The data showed the peptides with a higher hydrophilic value achieved higher labeling efficiency. We reasoned the peptides with shorter and higher hydrophilic values could be more easily dissolved in aqueous buffer, which resulted in higher reaction efficiency.

The 2-PCA labeling efficiency was reported to be impaired when the second position of peptide was proline. However, whether different amino acids on other positions could influence the labeling efficiency is still unknown. In this study, we systemically analyzed the influence of first three amino acids in the peptide on reaction efficiency. We found the reaction efficiency was higher when the first amino acid as L/A/E than others (Figures 2D,E, Supplementary Figures S1D,E). Perhaps, less steric hindrance (A/L) and more hydrophilicity (E) of the first amino acid (A) benefited the chemical conversion of 2-PCA coupling. Interestingly, our results showed glycine as the first amino acid supplied less steric hindrance but dramatically inhibited the “reaction efficiency”. Such lower reaction efficiency observed for peptides containing N-terminal glycine may be mainly due to the Thorpe–Ingold effect that led to kinetically slower cyclization (Kaneti et al., 2004). Our results showed proline as the second amino acid could inhibit the reaction, which was agreed with the previous study reported (MacDonald et al., 2015). In addition, we found lysine as the first amino acid could also affect the reaction efficiency. The iceLogo analysis indicated the peptides with first amino acid as A/E were overrepresented in fully labeled peptides (Figure 2F). These results indicate the 2-PCA labeling with high α-amino group labeling efficiency is applicable for the proteomic analysis.

### 2-PCA and biotin labeling efficiently blocks amino group on the peptide N-terminal and the lysine side chain

**FIGURE 3:**
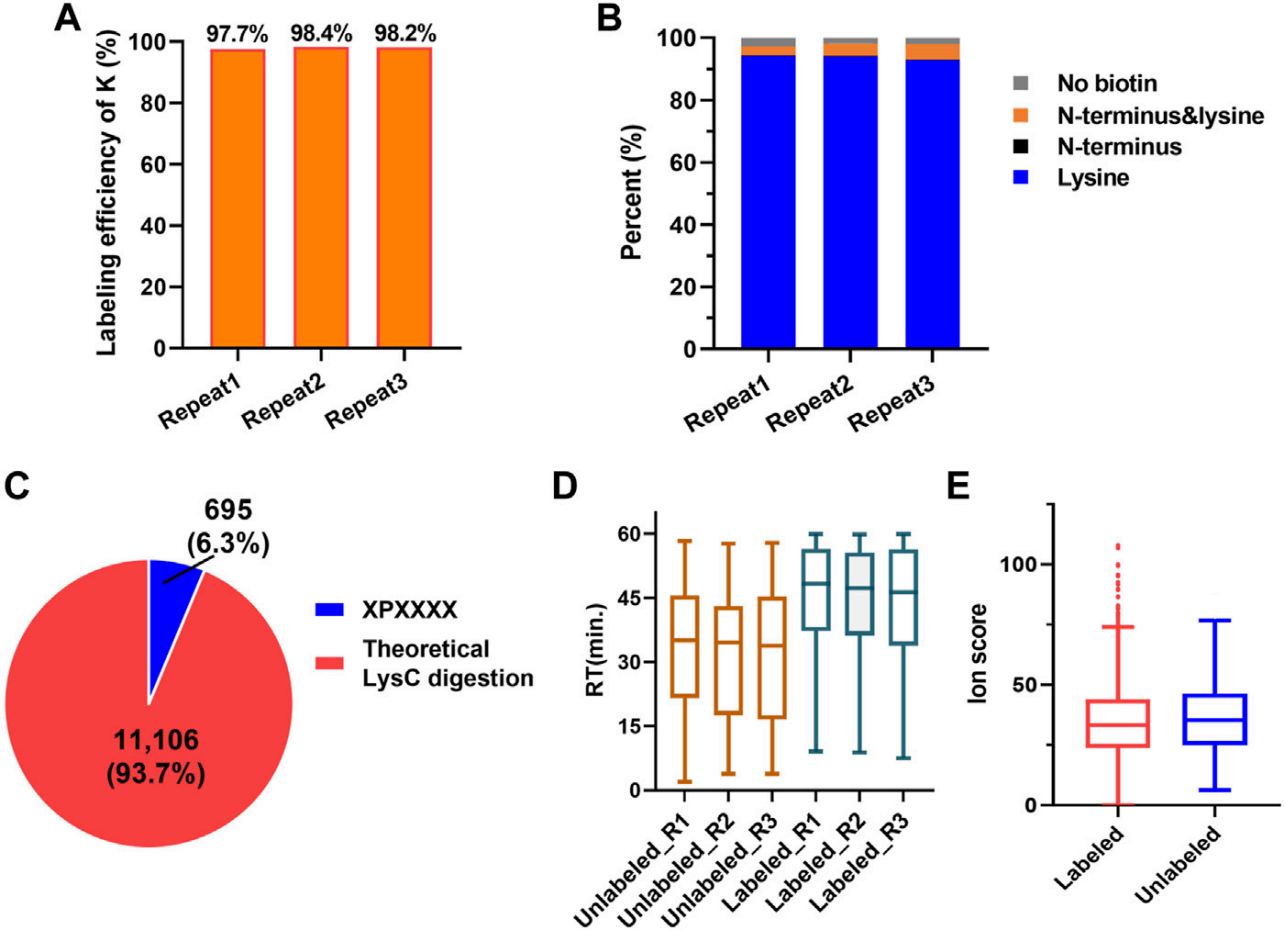
2-PCA and biotin labeling displays high-efficiency labeling for α-amino on peptide N-termini and ε-amino group on lysine. **(A)** Histogram distribution of biotin labeling efficiency on amino group in lysine in three technical replicates. More than 97.7% ε-amino group on K was blocked. **(B)** Histogram distribution of blocking efficiency on amino group in protein N-terminal in three technical replicates. More than 99.7% α-amino group on the peptide N-terminal were blocked. **(C)** Percentage of the theoretic “2-PCA-labeling” and “un-labeling” human protein C-terminal peptide by *in silico* digestion using LysC. Only 695 C-terminal peptides with second amino acid as proline could not be labeled by 2-PCA. **(D)** Peptide retention time comparison among before and after 2-PCA labeling. **(E)** Peptide ion score comparison among before and after 2-PCA labeling. All the experiments were performed in technical triplicates from the same started proteome sample.

According to the PBC strategy design, the blocking efficiency of the amino group on the peptide N-terminal and the lysine side chain is the key to highly efficient C-terminal peptide enrichment. In order to evaluate the amino group blocking efficiency of the peptide tandem reaction with 2-PCA and biotin, we performed the C-terminal peptide blocking experiments in technical triplicates from same started proteome. We found more than 99.7% α-amino group on the peptide N-terminal and more than 97.7% ε-amino group on K were blocked (Figures 3A,B, Supplementary Table S4). Interestingly, the peptides with second amino acid as proline that could not react with PCA were labeled with biotin, which would help to remove such peptide by streptavidin beads and increase the C-terminal peptide enrichment efficiency. In addition, we analyzed the whole human proteome digested with LysC *in silico* and found only 695 C-terminal peptides with second amino acid as proline, which account for 6.3% in all 11,106 C-terminal peptides (comprised of 6–50 amino acid residues) (Figure 3C). So, we reasoned the C-terminal peptide with proline as the second position amino acid was minimal, which largely would not influence C-terminome peptide profiling using the PBC strategy. Since the chemical derivatization of peptides will influence the chromatography separation and peptide scoring (reflecting the peptide identification confidence), the retention time and the ion score of the same peptide with and without 2-PCA labeling were systematically compared. We found that the retention time was significantly increased after labeling. The retention time distribution clearly showed that the labeled peptides were dominantly eluted in the later stage of the LC gradient (Figure 3D, Supplementary Figure S1F). We reasoned the 2-PCA molecule contains a pyridine ring structure, which would increase the peptide hydrophobic value after labeling. The peptide matching scores showed no significance difference between peptide with and without 2-PCA labeling (Figure 3E), which indicates the 2-PCA labeling would not influence the C-terminal peptides identification by our PBC strategy.

### PBC strategy represents the highest enrichment efficiency among the reported C-terminomic strategies

**FIGURE 4:**
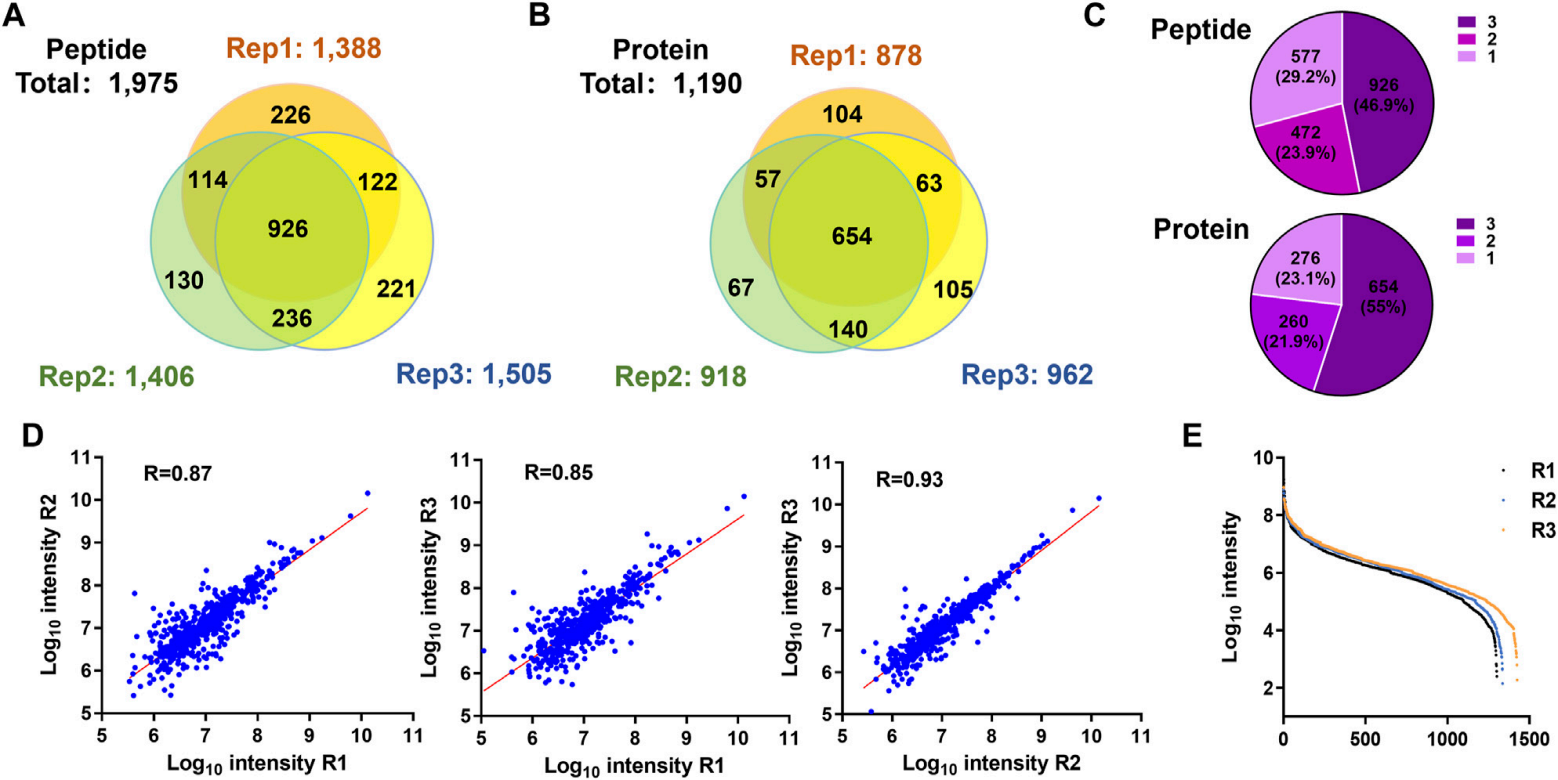
Highly efficient and high-throughput identification of protein C termini from 293T cells by using the PBC strategy. **(A,B)** Overlap analysis of C-terminal peptides **(A)** and proteins **(B)** from the triplicate experiment of C-terminal peptide enriched by using the PBC strategy. **(C)** Pie chart analysis of the frequency of C-terminal peptides identification from technical triplicates. A total of 46.9% peptides (926 peptides) were identified in all the triplicates, and 70.8% peptides (1,398 peptides) were identified in at least two replicates. **(D)** Correlation of intensity analysis of identified C-terminal peptides among technical triplicates. **(E)** Peptide abundance dynamic range of technical triplicates through the PBC strategy. The abundance dynamic range was larger than seven orders of magnitude.

We used the PBC strategy to enrich C-terminal peptides from cell sample and systematically evaluated its performance. In total, 300 µg proteins were used as the starting materiel, and three technical replicates were performed. Finally, we identified an average of 1,433 C-terminal peptides belonging to 919 proteins in each experiment (Figures 4A,B, Supplementary Table S5). It should be noted that our results showed a lot of C-terminal peptides were not blocked by 2-PCA at N-termini. We systematically evaluated the hydrophilic/hydrophobic values and peptide lengths of the enriched C-terminal peptides. We found the unlabeled C-terminal peptides displaying significantly higher hydrophobic values and peptide lengths than the 2-PCA–labeled or partially labeled C-terminal peptides (Supplementary Figure S2). These results were all consistent with our conclusions that the efficiency of 2-PCA labeling was related to the peptide hydrophilic/hydrophobic value in Figure 2C. A total of 1,975 C-terminal peptides belonging to 1,190 proteins were identified, which achieved 180% more C-terminal peptide identification than the highest reported C-terminal peptide number based on the chemical derivatization–based C-terminomics study. (Hu et al., 2019) We found that 46.9% peptides (926 peptides) were identified in all the triplicates, and 70.8% peptides (1,398 peptides) were identified in at least two replicates (Figure 4C). We also evaluated the quantification linear correlation among the replicates and found the Pearson correlation coefficients of each two pair among the three replicates were all remarkably high (>0.85) (Figure 4D). These results indicated that the PBC strategy provided a high-throughput and reproducibility for C-terminal peptide enrichment. The abundance dynamic range of the enriched C-terminal peptides from the PBC strategy was larger than seven orders of magnitude (Figure 4E). We compared our results with published C-terminome datasets of human cells (Table 1, Supplementary Figure S3A). Our PBC strategy achieved the highest identification number of C-terminal peptides among all reported chemical derivatization–based strategies. In addition, the C-terminal peptide enrichment efficiency of our PBC method is 68% (the percent of C-terminal peptides among all identified peptides), which is highest in the C-terminomics study up to date. Thus, although the enriched C-terminal peptides were not completely labeled with 2-PCA, the PBC provides a novel and simple strategy for high efficiency and reproducibility C-terminal peptides enrichment.

**TABLE 1:** Comparison the results of human protein C-terminomics studies.

#	This study	Hu et al. (2019)	Li et al. (2020a)	Wang et al., (2021)
Cell type	293 T	293 T	293 T	HeLa
Sample amount	900 µg	1.5 mg	120 µg	1 mg
Enzyme	LysC	LysargiNase	LysargiNase	Trypsin
Number of C-terminal peptides	1,975	1,100	2,000	4,724
Selective enrichment efficiency (%)	68	39.1	22.4	2.7–34
Number of protein C-termini	1,190	924	1,812	2,219
Isolation method	Two chemical reaction steps	Three chemical reaction steps	Three chemical reaction steps + SCX	SCX

### Overview of C-terminome on 293 T cells

**FIGURE 5:**
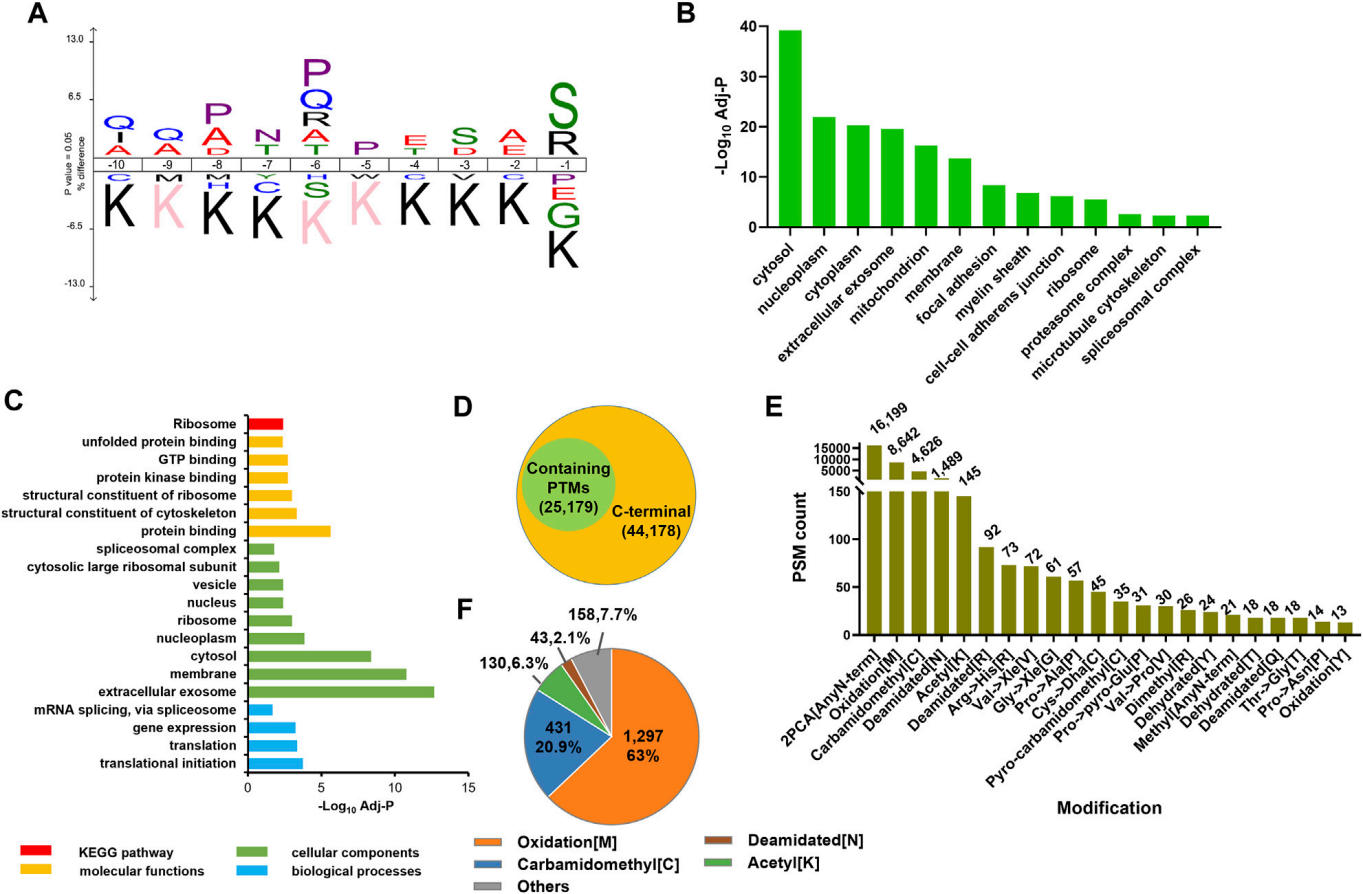
Function analysis of the enriched C-terminal truncated proteins and the PTM distribution on C-terminal peptide. **(A)** IceLogo analysis of the last 10 amino acids of original C-terminal identified by PBC strategy. **(B)** Cellular components enrichment analysis of C-terminal proteins identified by PBC strategy. **(C)** The gene ontology and KEGG pathway enrichment analysis of the proteins with C-terminal truncated. The truncated proteins significantly enriched in extracellular exosome, vesicle, and ribosome. **(D)** Pie chart analysis of identified PSM with and without containing PTMs. In total, more than 30% PSM were identified with PTM-labeled peptides. **(E)** Distribution of PTMs modified on C-terminal peptide. **(F)** Distribution of identified PTMs on last amino acid of protein C-termini.

The C-terminomic study could construct the landscape of C-termini in cell with high throughput and efficiency, which could be used to study the C-end rules in cells with high accuracy. In our study, the iceLogo analysis of the C-terminal dataset showed that the P/Q/S is highly enriched in the protein C-terminal sequence (Figure 5A). This result was consistent with our previous study using the LAACter method. (Hu et al., 2019) In addition, the lysine was observed under-represented at −10 to −1 position, which could be explained by that the peptide sample used in the PBC strategy was lysC digestion generated. In order to study whether the C-terminal peptide enrichment bias existed in our PBC strategy, gene ontology enrichment was used to systematically evaluate our C-terminal dataset. We found the enriched C-terminal peptides distributed among different cellular compositions (Figure 5B, Supplementary Table S6), such as cytosol, membrane, ribosome, and nuclear. “C-end rules” was reported as the key factor influencing protein functions in cell (Lin et al., 2018). In addition, we also systemically compared the distribution of first three amino acids in enriched C-terminal peptide and theoretical C-terminal peptide. The theoretical C-terminal peptides were generated from *in silico* digestion of whole human proteome by using LysC (Figure 3C). The results showed the similar distribution of first three N-terminal amino acids between enriched and theoretical C-terminal peptide (Supplementary Figures S3B–D), which also indicated the PBC strategy could enrich C-terminal peptides without bias.

Our C-terminomic study enabled us to examine C-terminal truncated proteins (Supplementary Table S7). Totally, 217 C-terminal truncation peptides corresponding to 50 proteins were identified. For example, six truncation peptides were identified from HSP90AB1 (Table 2). The annotated MS/MS spectra of these six truncation peptides are showed in Supplementary Figure S3E. In addition, we also found the truncated histone protein H4 in our data (Table 2). The annotated MS/MS spectra of the truncation peptides of H4 are showed in Supplementary Figures S4A,B. In order to further analyze the cellular composition and molecular functions of these 50 truncated proteins, the gene ontology (GO) and KEGG pathway enrichment were performed (Figure 5C). We found these proteins significantly enriched in extracellular exosome, vesicle, and ribosome, suggesting these proteins mainly partook in the biological processing of translation and translational initiation. The ribosome pathway was also significantly enriched through the KEGG pathway analysis. We inferred the truncated proteins were mainly generated from two origins. One was from immature proteins due to the incomplete translation step in ribosome; another might come from the proteolytic fragments by exopeptidases and metallocarboxypeptidase enzyme in exosome or vesicle.

**TABLE 2:** Identified Neo-N-termini for HSP90AB1 (P08238) and H4 (P62805).

Gene name	Protein accession number	Annotated sequence
HAP90AB1	P08238	[K].LGLGIDEDEVAA.[E]
		[K].LGLGIDEDEVAAEEPNAAVPD.[E]
		[K].LGLGIDEDEVAAEEPNAAVPDEIPPLEGD.[E]
		[K].LGLGIDEDEVAAEEPNAAVPDEIPPLEGDED.[A]
		[K].LGLGIDEDEVAAEEPNAAVPDEIPPLEGDEDASR.[M]
		[K].LGLGIDEDEVAAEEPNAAVPDEIPPLEGDEDASRMEEVD.[-]
H4	P62805	[K].VFLENVIR.[D]
		[K].VFLENVIRD.[A]
		[K].VFLENVIRDA.[V]
		[K].VFLENVIRDAV.[T]
		[K].VFLENVIRDAVTY.[T]
		[K].RQGRTLYGF.[G]
		[K].RQGRTLYGFG.[G]
		[K].RQGRTLYGFGG.[-]

PTMs on protein C-terminal region play important roles in protein functions (Marino et al., 2015). However, the reported PTMs studies on C-terminal were limited due to the lack of in-depth and large-scale C-terminome data. In this study, we used the open-search method to analyze the PTMs distribution in our C-terminomic dataset. All the raw data were reanalyzed by using pFind 3.1 search engine through open-search searching mode. Totally 44,178 PSMs were identified and more than 30% PSM were identified with containing PTMs (Figure 5D, Supplementary Table S8). The PTM types and their frequency distributions is showed in Figure 5E. The dominant PTM was 2-PCA modification on peptide N-terminus, which was introduced by chemical divinization *in vitro* at the peptide level. Deamidation on asparagine was frequently occurred in our data. Deamidation was reported could occur spontaneously on proteins both *in vivo* and *in vitro* (Hao et al., 2011; Brown et al., 2017). *In vitro*, the deamidation rate of protein or peptides closely related with pH and temperature of sample preparation buffer. In our PBC method, the peptides were reacted with 2-PCA and biotin all performed at pH 8.5 under 37°C. We reasoned that such high frequency of deamidation were mainly artefacts introduced in sample preparation. Amino acid substitutions were reported to alter physiological properties of protein (Huang and Gromiha, 2010; Gromiha et al., 2019), such as the enzymatic stability and protein folding rate. We found the acetylation on lysine and amino acid substitution highly occurred in the protein C-terminal region (Figures 5E,F). Interestingly, we also found many lysines at protein C-termini with acetylation, which was also reported benefit for protein stability (Wang et al., 2017). In addition, we also found 176 PSMs were identified as the PTM-labeled histone peptides (Supplementary Figure S4C). The main PTM types were deamination, amino acid substitution, and dehydration. Thus, the PBC strategy provides a high-throughput analysis of the PTMs on the protein C-terminal region.

## Conclusion

Due to the high complexity of peptidome sample, the signal of C-terminal peptide could easily be suppressed by other internal peptides in LC-MS detection. Protein C-terminal peptide profiling is still challenging. In this study, we developed 2-PCA- and biotin-labeling based C-terminomic (PBC) proteomic strategy to globally profile C-terminal peptides. We first systemically investigated the characters of 2-PCA labeling on complex peptide sample by using the proteomic method and found the peptide length, peptide hydrophilic value, and first amino acid composition significantly influenced the reaction efficiency. Importantly, the peptidome-wide labeling efficiency and selectivity of 2-PCA sufficiently high for labeling peptide N-terminal amines. The PBC strategy used LysC to digest proteome and only two chemical derivatization steps for C-terminal peptide profiling, which is less labor- and time-consuming than current reported C-terminal peptide profiling. According to the PBC-based C-terminomic results, a total of 1,975 C-terminal peptides belonging to 1,190 proteins were identified. This strategy showed the highest C-terminal enrichment efficiency among all the reported strategies. Our C-terminome results also revealed neo-C-terminal on proteins and new PTMs on C-terminal peptides, which would help in-depth study of the protein C-terminome and uncover more characters and biological functions of protein C-termini.

It should be noted that few limitations exist in the PBC technology. First, the 2-PCA reaction could not completely block all the α-amino groups on peptide N-termini, which lead to sub-optimal 2-PCA labeling on C-terminal peptides in PBC method. However, the technology evaluation results showed that 80% of the C-terminal proteins were identified in at least two technical replicates, and the correlation of the enriched C-terminal peptides was higher than 0.85 (Figures 4C,D), which suggest that this method is reliable and robust for the enrichment of C-terminal peptides. Second, 6.3% C-terminal peptides in the whole proteome contain proline as the second amino acid in theory, which could not react with 2-PCA and be enriched by PBC method. Nonetheless, the PBC method provides a novel idea for C-terminal peptide enrichment based on combination of α-amino group blocking, lysC digestion, and negative selection. In addition, since C-terminal peptides are dominant in the enrichment peptides and with low background interference, the PBC method can be combined the label free quantification technology or carboxyl group selectively isotope labeling technology to realize the quantitative analysis of C-terminal peptides. For example, combining with isotope labeling quantification technology by using the isotope labeling reagent d_0_-/d_6_-2,4-dimethoxy-6-piperazin-1-yl pyrimidine (DMPP), which could label the carboxyl group on peptide with high efficiency and selectivity (Leng et al., 2013). In addition, the PBC method could be also further simplified and optimized into one step chemical derivatization for C-terminal peptide enrichment by combining with commercially available hyperbranched polyaldehyde polymers (HPG-ALD polymers) (Supplemntary Figure S5) (Kleifeld et al., 2010; Kleifeld et al., 2011). After PCA labeling, the protein N-terminal peptides and internal LysC-digested peptides could be depleted through HPG-ALD polymers directly to enrich the C-terminal peptides. The simplified method by HPG-ALD polymers is devoid of further biotinylation on lysine and using streptavidin beads to deplete non-C-terminal peptides. To sum up, despite the enriched C-terminal peptides were not completely labeled with 2-PCA, the PBC provides a novel and simple strategy for high efficiency and reproducibility of C-terminal peptides enrichment.

## Data Availability

The datasets presented in this study can be found in online repositories. The names of the repository/repositories and accession number(s) can be found in the article/Supplementary Material.

## Supplementary material

SUPPLEMENTARY FIGURE S1 The evaluation for 2-PCA labeling efficiency in LysC-digested peptides. The number of protein **(A)** and peptide **(B)** identification before and after 2-PCA labeling. **(C)** The 2-PCA labeling efficiency analysis of α-amino group on peptide N-terminal and ε-amino group on lysine. **(D,E)** The identified number distribution of full labeling **(D)** and un-labeling **(E)** peptide on different first three amino acids. **(F)** The histogram distribution of peptide retention time before and after labeling with 2-PCA.

SUPPLEMENTARY FIGURE S2 The influences of 2-PCA labeling efficiency analysis of C-terminal peptides. The hydrophilic/hydrophobic value **(A)** and peptide length **(B)** comparison of 2-PCA fully-labeled, partially labeled, and unlabeled C-terminal peptides. The p-value was calculated by paired Student *t*-test.

SUPPLEMENTARY FIGURE S3 The analysis of C-terminome data by using the PBC method. **(A)** Overlap analysis of our C-terminome data with other published data. **(B,C,D)** The distribution comparison of the first amino acid **(B)**, second amino acid **(C),** and third amino acid **(D)** between enriched C-terminal peptide and theoretical C-terminal peptide. The theoretical C-terminal peptide was generated from *in silico* digestion of whole human proteome by using LysC. **(E)** The MS/MS spectrum for truncated C-terminal peptides from HSP90AB1.

SUPPLEMENTARY FIGURE S4 The analysis of truncations and PTMs on histone.

SUPPLEMENTARY FIGURE S5 The further optimized PBC method. The PBC method could be simplified and optimized into one-step chemical derivatization for C-terminal peptide enrichment. After PCA labeling, the protein N-terminal peptides and internal LysC peptides could be depleted through commercially available hyperbranched polyaldehyde polymers (HPG-ALD polymers) directly and the C-terminal peptides were enriched.
