# Supplementary material for: PBC, an easy and efficient strategy for high-throughput protein C-terminome profiling
Source: Front Cell Dev Biol. 2022 Aug 31;10:995590. doi: 10.3389/fcell.2022.995590 (PMC9471192; doi:10.3389/fcell.2022.995590)

Supplementary Figure 1

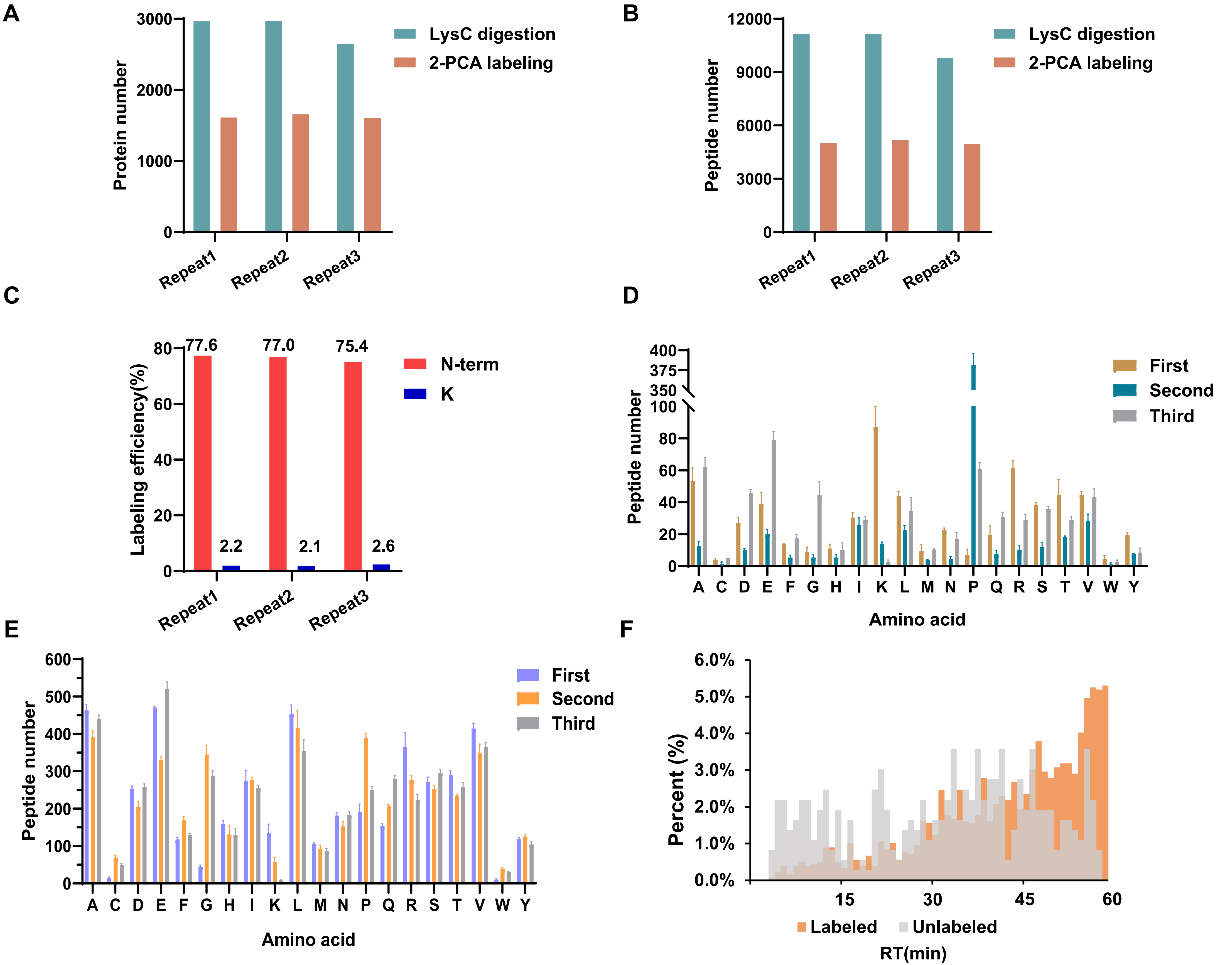

Supplementary Figure 2

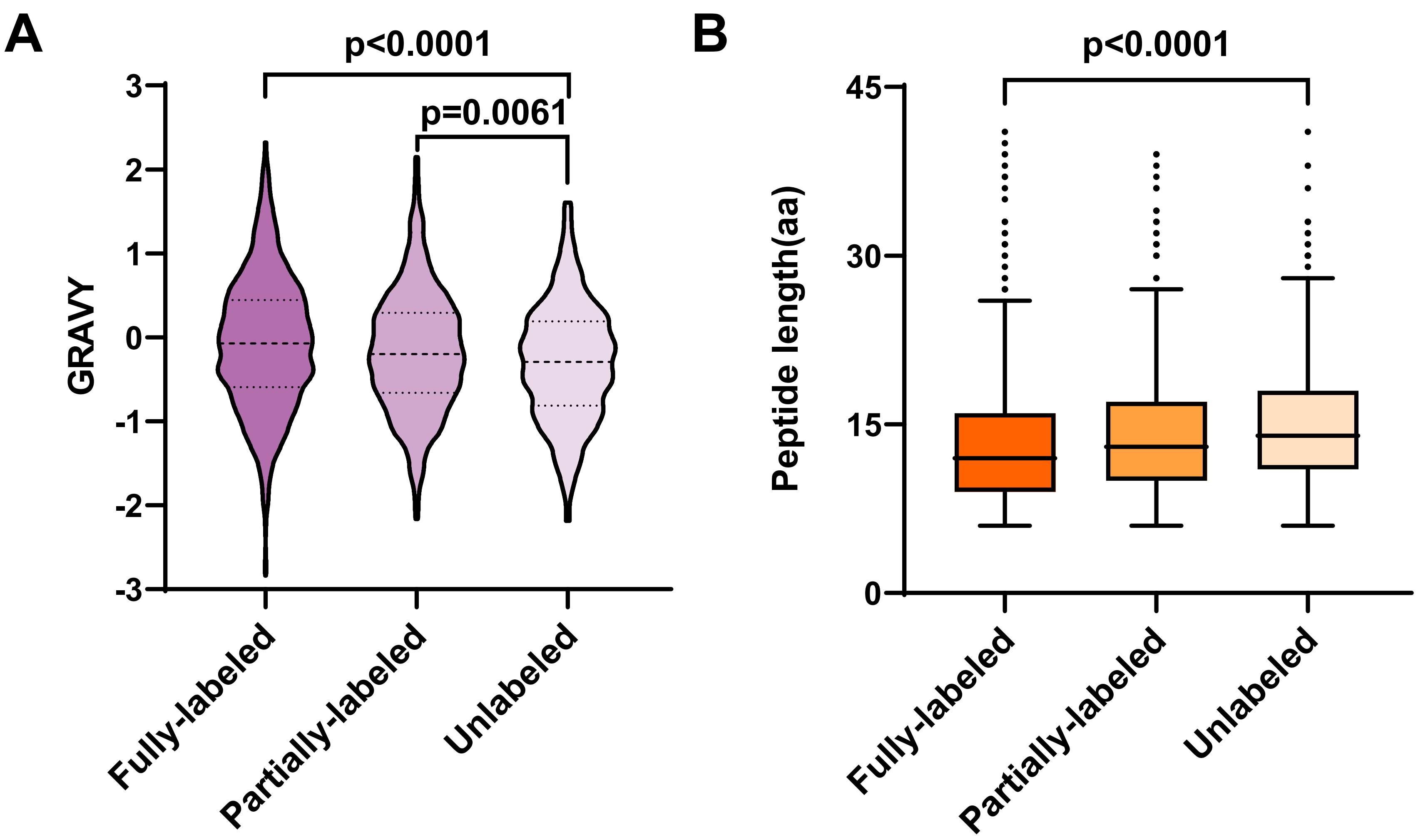

Supplementary Figure 3

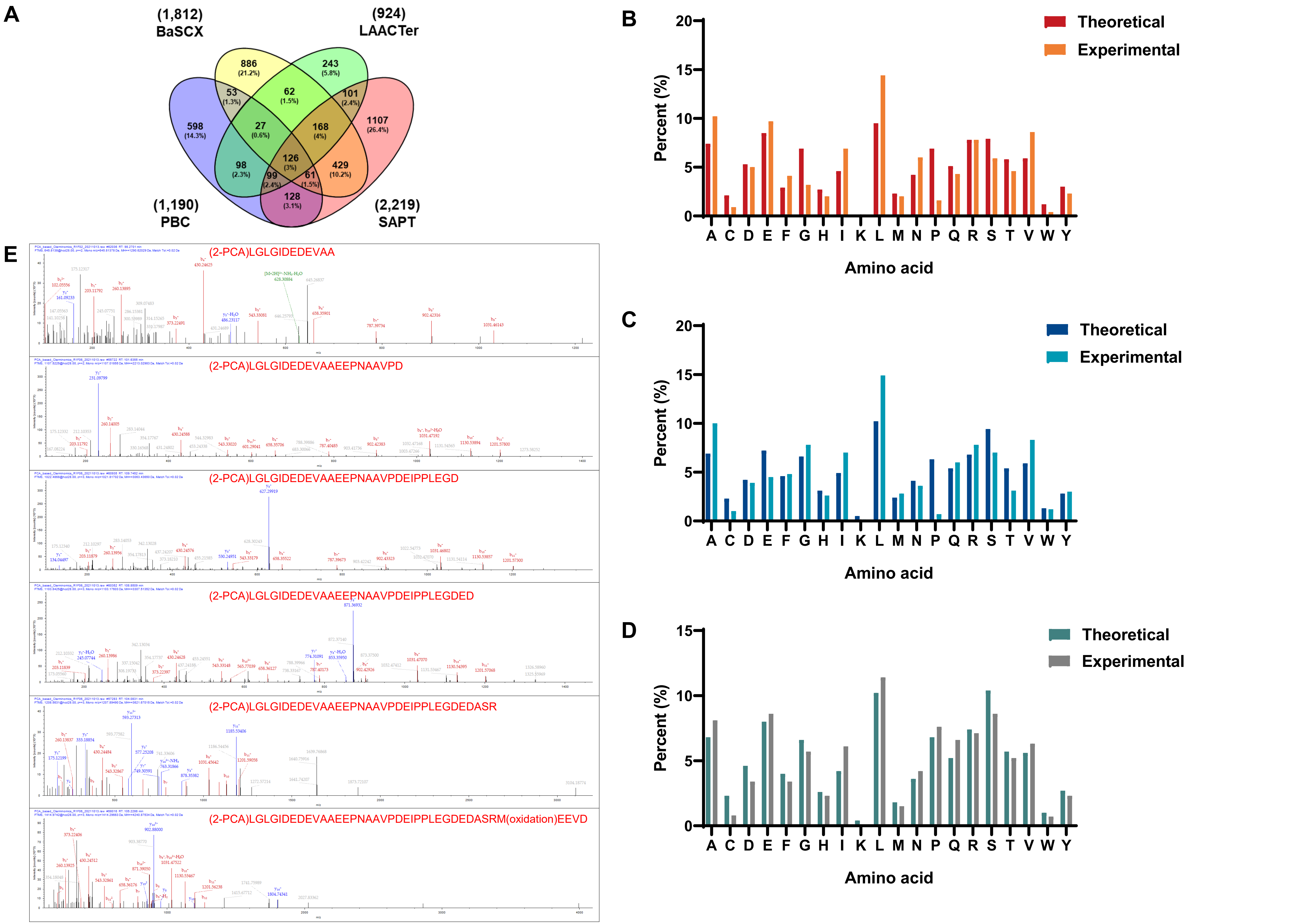

Supplementary Figure 4

A

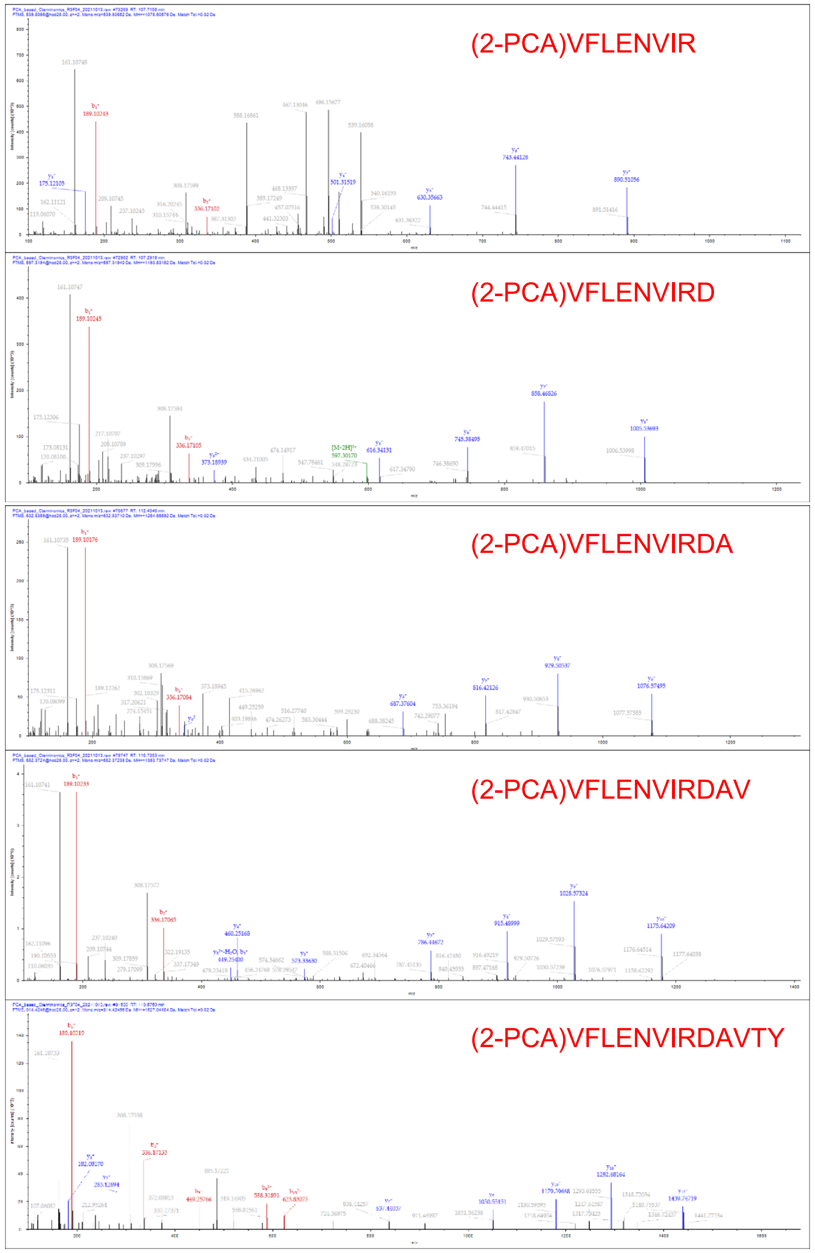

B

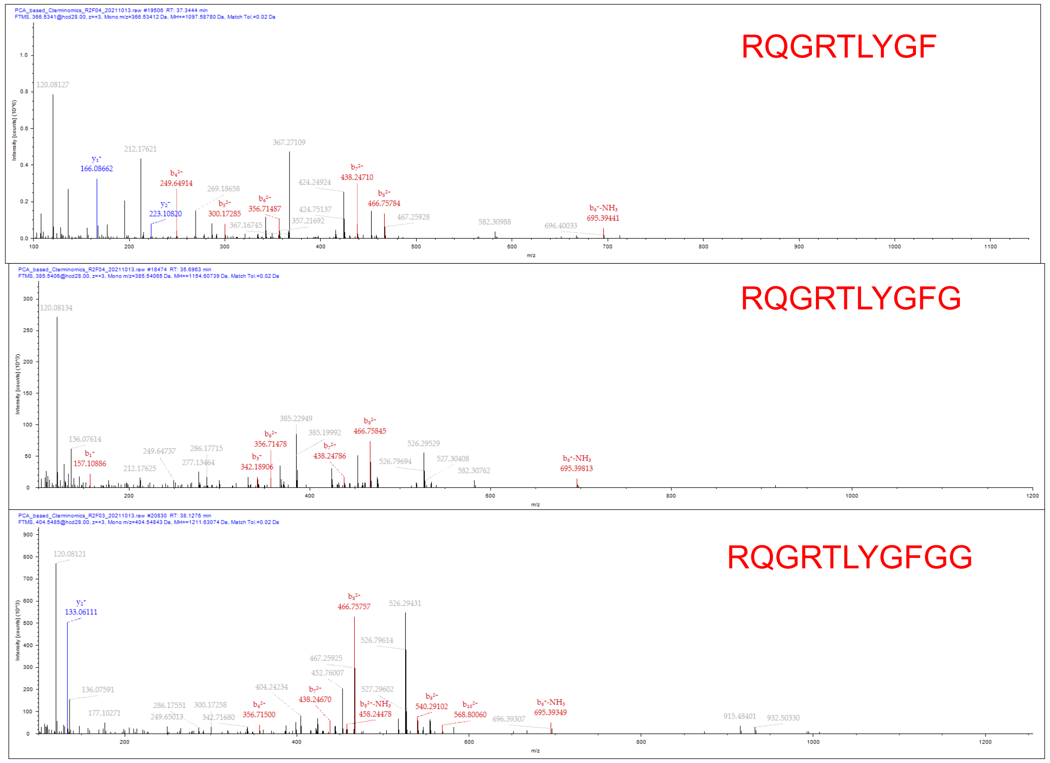

C

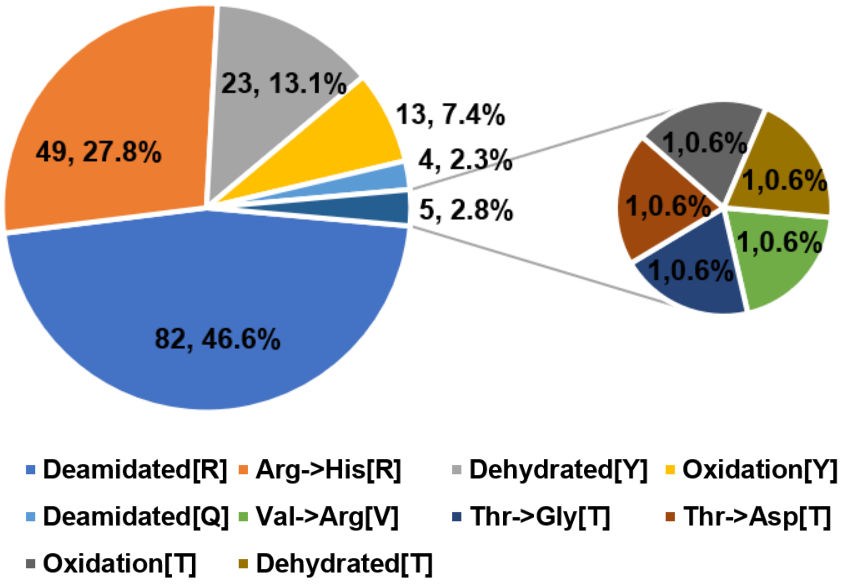

Supplementary Figure 5

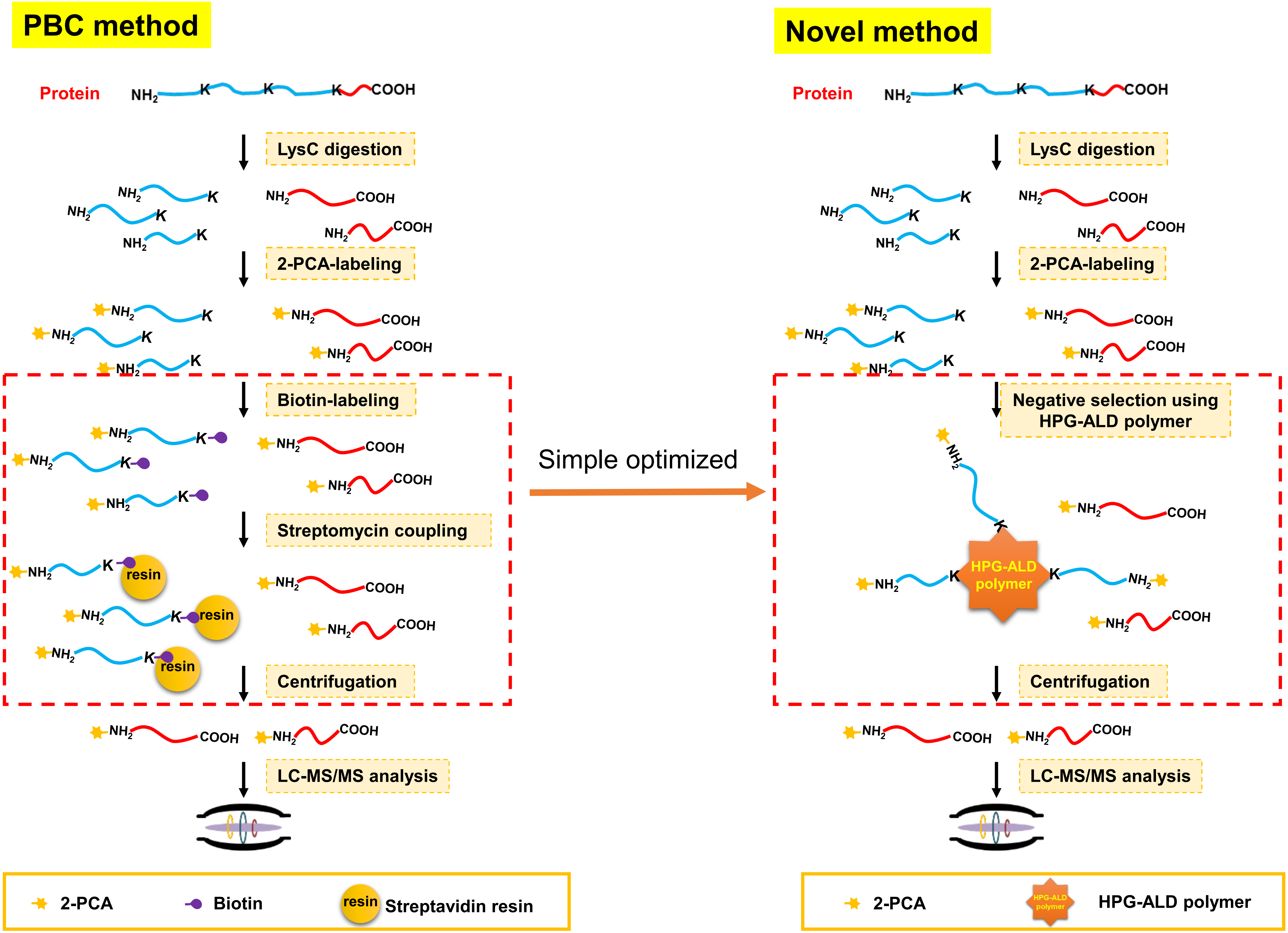

Supplement: Supplementary file 2 [file DataSheet1.PDF]
